# The Bearing Characteristics of a Grillage Root Foundation Based on a Transparent Soil Material: Enhancing the Bearing Capacity

**DOI:** 10.3390/ma18071470

**Published:** 2025-03-26

**Authors:** Zehui Ma, Junjie Wang, Xuefeng Huang, Zhifeng Ren, Hao Wang

**Affiliations:** 1 School of Civil Engineering, Chongqing Jiaotong University, Chongqing 400074, China; 2 School of Materials Science and Engineering, Chongqing Jiaotong University, Chongqing 400074, China; 3 College of Architecture and Energy Engineering, Wenzhou University of Technology, Wenzhou 325000, China; 4 China Merchants Zhongyu Engineering Consulting (Chongqing) Co., Ltd., Chongqing 400067, China

**Keywords:** transmission tower foundation, transparent soil, model testing, prediction of bearing capacity, root, pile–soil interaction, Elman neural network, machine learning optimization

## Abstract

The construction of a power grillage is of great significance for promoting local economic development. Identifying the characteristics of foundation damage is a prerequisite for ensuring the normal service of the power grillage. To investigate the bearing mechanism and failure mode of the grillage root foundations, a novel research method with a transparent soil material was used to conduct model tests on different types of foundations using particle image velocimetry (PIV) technology. The results indicate that, compared to traditional foundations, the uplift and horizontal bearing capacities of grillage root foundations increased by 34.35% to 38.89% and by 10.76% to 14.29%, respectively. Furthermore, increasing the base plate size and burial depth can further enhance the extent of the soil displacement field. Additionally, PIV analysis revealed that the roots improve pile–soil interactions, transferring the load to the surrounding undisturbed soil and creating a parabolic displacement field during the uplift process, which significantly suppresses foundation displacement. Lastly, based on experimental data, an Elman neural network was employed to construct a load-bearing capacity prediction model, which was optimized using genetic algorithms (GAs) and the whale optimization algorithm (WOA), maintaining a prediction error within 3%. This research demonstrates that root arrangement enhances the bearing capacity and stability of foundations, while optimized neural networks can accurately predict the bearing capacity of grillage root foundations, thus broadening the application scope of transparent soil materials and offering novel insights into the application of artificial intelligence technology in geotechnical engineering. For stakeholders in the bearing manufacturing industry, this study provides important insights on how to improve load-bearing capacity and stability through the optimization of the basic design, which can help reduce material costs and construction challenges, and enhance the reliability of power grillage infrastructure.

## 1. Introduction

The design of pile foundations in geotechnical engineering is the core link to ensuring structural stability and safety [1], especially in complex geological conditions [2] or harsh environments [3,4]. The bearing performance of pile foundations directly determines the overall reliability of the project [5,6]. Due to the complexity and opacity of natural soil, in traditional pile foundation model tests it is difficult to visually observe the evolution of displacement fields inside the soil and the pile–soil interaction mechanism, which limits a deeper understanding of failure mechanisms. Therefore, scholars have been exploring alternative materials that can simulate the characteristics of natural soil and are easy to observe. As a new type of experimental material, transparent soil has gradually attracted attention and has been widely used in geotechnical engineering research in recent years [7]. The aim is to achieve soil transparency through the refractive index matching of fused silica sand and specific pore liquids [8]. This is followed by integrating particle image velocimetry (PIV) technology to visually observe the internal displacement field within the soil, which shares similarities with the experimental methodologies employed in waste treatment research. Both involve meticulous control over material compositions and process conditions to achieve the desired physical and chemical properties [9]. Research on new transparent soil materials is not only limited to the preparation of materials and the exploration of their physical and mechanical properties [10,11], but also gradually expands to their simulation and analysis in different engineering applications [12,13,14,15]. The preparation technology for transparent soil is continuously being optimized and, in terms of material ratio, Carvalho [16] introduced a new low-viscosity transparent soil formula, which has the main advantages of a lower viscosity, a lower sensitivity to temperature variations, and the ability for the materials to be recycled for use in multiple tests. In addition, in the field of pile foundation engineering, transparent soil provides a powerful tool for studying the interaction mechanism between piles and soil, and transparent soil technology is widely used to analyze the bearing mechanism of different pile types. Through transparent soil model tests, the displacement field changes in the soil around the pile can be visually observed, and the interaction mechanism between the pile and the soil for different pile types and loading conditions can be analyzed. Xiao [17] presents model tests on the displacement characteristics of flat-ended piles, pipe piles, and piles with a cone tip of 45 degrees in a transparent granular soil. Ding [18] developed a new soil-arching effect test device and its testing method uses transparent soil method and particle image velocimetry technology. In the work by Sang [19], based on a transparent soil material, a model test was carried out on single piles with and without pile caps and the influence of pile cap–soil interactions on the load transfer mechanism of the pile foundations was studied. Liu [20] intuitively demonstrated, through transparent soil tests, that the different penetration mechanisms of flat-end piles and tapered-end piles are mainly due to the formation of sub stable sand plugs below the pile tip. Existing research has mostly focused on traditional pile types, and there is still a lack of systematic research on the bearing characteristics and displacement field distribution of new grillage root foundations using transparent soil materials. In particular, the influence of the number of roots on the bearing capacity and displacement field of the piles is still unclear.

The optimization of pile foundation bearing performance has consistently been a central topic in geotechnical engineering research. Traditional pile foundations enhance their bearing capacity by increasing either the pile diameter or burial depth; however, these methods are constrained by material costs and construction challenges. In recent years, the design of root bond structures has garnered significant attention due to their ability to substantially improve pile–soil interactions [21,22,23,24]. Roots effectively disperse loads and mobilize a broader range of soil to participate in stress distribution by expanding the surface area of the pile and embedding it into deeper soil layers. Specifically, root structures serve as “bridges”, connecting the pile foundation to the soil. When a load is applied to the top of the foundation, the roots disperse the load into a broader area of the surrounding soil through their frictional interaction with the soil, fully utilizing the excellent shear strength of the adjacent undisturbed soil. This results in a more uniform and stable stress distribution. This dispersion mitigates stress concentrations near the foundation top, thereby enhancing the overall stability of the foundation. Zhou [25] demonstrated the advantages of reducing pile-top displacement by comparing the numerical simulation results of the load–displacement relationship and the bearing capacities corresponding to different variables. Additionally, grillage foundations [26,27] have emerged as a research hotspot due to their modular design and ease of construction; however, their load transfer paths and failure modes require further verification through transparent soil tests. With the rapid advancement of artificial intelligence technology, its application in geotechnical engineering for the prediction, monitoring, and optimization of soil’s mechanical properties has begun to take shape [28,29]. By analyzing extensive experimental data, AI can elucidate complex physical phenomena and provide precise guidance for engineering practice. Nevertheless, challenges remain in employing machine learning to predict the bearing capacity of pile foundations, particularly regarding insufficient model generalization under small sample conditions. The application of existing artificial intelligence technologies in transparent soil research is still nascent and has not fully leveraged its advantages in data processing and prediction optimization. Therefore, integrating transparent soil research with artificial intelligence technology can enhance both the accuracy and efficiency of transparent soil testing while expanding its applications in geotechnical engineering, yielding significant scientific and practical value.

In response to these issues, this article focuses on grillage root foundations as the primary research subject. By integrating transparent soil model tests with artificial intelligence technology, we aim to conduct an in-depth analysis of their bearing mechanisms and failure modes. The specific research questions include: (1) How does the load–displacement curve indicate that the implantation of roots significantly enhances the bearing capacity of the foundation, and how does the grillage root foundation effectively transfer and disperse the load through the interaction between the roots and the soil? (2) Through transparent soil model tests, we will observe the displacement field changes in grillage root foundations of varying sizes and with different numbers of roots during the loading process, and analyze the influence of roots on the foundation’s bearing capacity. (3)Additionally, we will utilize genetic algorithms (GAs) and the whale optimization algorithm (WOA) to optimize the Elman model for small sample sizes, thereby improving the model’s prediction accuracy and generalization ability.

## 2. Model Experiments

### 2.1. Model Test Equipment

**Figure 1:**
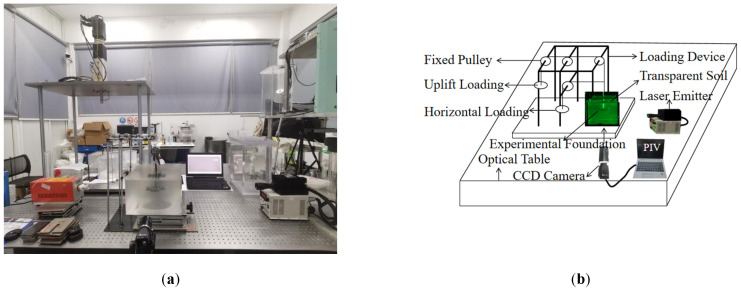
Indoor installation diagram: (**a**) indoor drawing; (**b**) schematic drawing.

The indoor transparent soil testing system primarily comprises a testing platform, a laser emitter, a camera, a loading rack, a model box, transparent soil, model piles, and a particle image velocimetry (PIV) measurement system. The layout of the model test is illustrated in Figure 1. This experiment employs a fixed pulley system for loading, with the loading apparatus consisting of an anti-seismic loading platform, a fixed pulley system, nylon wire, and weight plates. During the experiment, the nylon line consistently aligns with the centerline of the foundation to ensure that the foundation’s geometric center is loaded. The data acquisition system includes high-frequency lasers, CCD (Charge-Coupled Device) high-speed industrial cameras (model:Lt425C, Teledyne Lumenera, Canada), and PIV data processing software (PIVview2). The camera is set to take timed shots to avoid shaking caused by human photography and to ensure that the speckle field image is sufficiently stable. The laser model utilized is the DR-532CP-5 sheet-like optical laser, which has a test power of 2.4 W.

### 2.2. Preparation and Principle of Transparent Soil

The experiment utilized baked quartz sand with a particle size distribution ranging from 0.5 to 1.0 mm to simulate transparent soil particles. This sand has a specific gravity of 2.186, a refractive index of 1.4585, and minimum and maximum dry densities of 0.970 g/cm^3^ and 1.274 g/cm^3^, respectively, and is provided by Wanhe Mining Co., Ltd. (Xinyi, China). The internal friction angles of the dry and oily samples, obtained from the direct shear test, were 37.3° and 38.3°, respectively. The materials for simulating the pore liquid were C_12_H_26_ (dodecane), manufactured by Tianjin Kemio Chemical Reagent Co., Ltd. (Tianjin, China), and No 15 white oil, the manufacturer of which is Maoming Hongtai Petrochemical Co., Ltd. (Manming, China). During the experiment, C_12_H_26_ and No. 15 white oil were mixed in a mass ratio of 1:4 and verified using an Abbe refractometer. The quantities of both liquids were fine-tuned to adjust the refractive index to 1.4585 at a temperature of 24 °C. Since the refractive index of the simulated soil mass (fused silica sand) and pore fluid (the mixture of C_12_H_26_ and No. 15 white oil) is 1.4585, light does not refract between the soil mass and the pore fluid, rendering the soil mass transparent. The specific operational steps are as follows:

(1) The mixed liquid, primarily consisting of white oil and C_12_H_26_, must have a refractive index that matches that of the fused silica sand.
(2) Pour the mixed liquid into the container, then slowly add the pre-prepared mixed solute while continuously stirring to ensure that the fused silica sand is fully immersed in the mixed liquid, thereby minimizing the formation of bubbles in the transparent soil.
(3) Place the prepared transparent sand in a vacuum chamber and subject it to vacuum treatment at −0.1 MPa for 1 h. Maintain the vacuum environment to allow the transparent sand to settle within the chamber, ensuring the complete elimination of bubbles.
(4) Transfer the vacuum-treated transparent sand into the experimental system and allow it to stand until the soil is fully consolidated.

The fundamental principle of the transparent soil model test involves preparing transparent soil in a model box and securing the model foundation. In a dark environment, the model foundation is illuminated with a laser emitter to create a speckle field. The foundation is loaded using a loading frame and weights. An electronic displacement meter is fixed to the top of the foundation via a magnetic gauge holder to measure the displacement during the test. Throughout the process, a camera records changes in the speckle field. Particle image velocimetry (PIV) software is employed to analyze a series of laser speckle field images obtained during the experiment. The software divides the images into several equally sized blocks, matches the blocks of the altered image with those of the initial image, and uses correlation analysis to determine the positions of the altered image blocks within the initial image, thereby obtaining the displacement of each block. This matching process is repeated for the remaining blocks, and the displacement direction and magnitude of the altered image are represented using a vector diagram. Finally, the complete displacement field of the soil surrounding the foundation under load is obtained.

### 2.3. Production of Model Boxes and Model Piles

**Figure 2:**
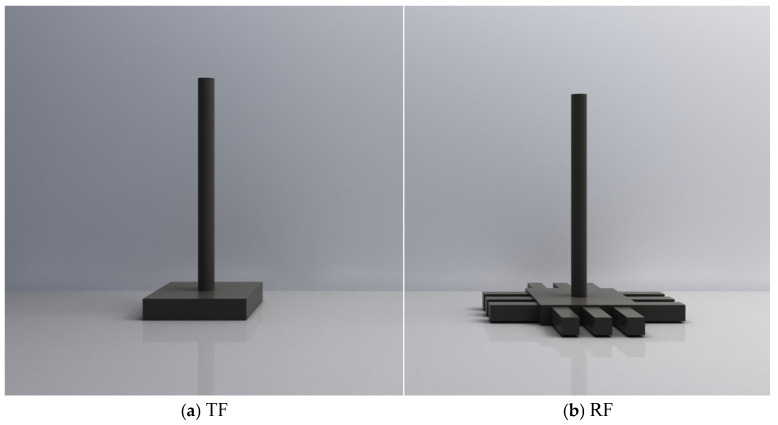
Model Foundation. (**a**) TF; (**b**) RF.

The model box is constructed from 4 mm thick transparent acrylic and is shaped as a rectangular prism with an open top. Its dimensions are 240 mm in length, 240 mm in width, and 200 mm in height. The model pile is designed using SolidWorks software (SolidWorks 2025), as illustrated in Figure 2. It is 3D printed from a gray graphite nylon material, resulting in a total of six model foundations—three grillage foundations and three grillage root foundations. These are used to compare and analyze the differences in soil displacement fields between traditional and root foundations. The model pile is positioned at the center of the model box, with its base 60 mm above the bottom of the transparent soil. To simulate a rough contact surface, a layer of quartz sand is bonded to the model foundation. Firstly, apply the adhesive, mainly composed of cyanoacrylate ethyl ester, evenly on the surface of the pile, after which the foundation is horizontally rotated, and quartz sand is uniformly sprinkled over the pile surface. Once the quartz sand is securely bonded, the surface is gently brushed to remove excess particles.

### 2.4. Model Test Procedure

**Figure 3:**
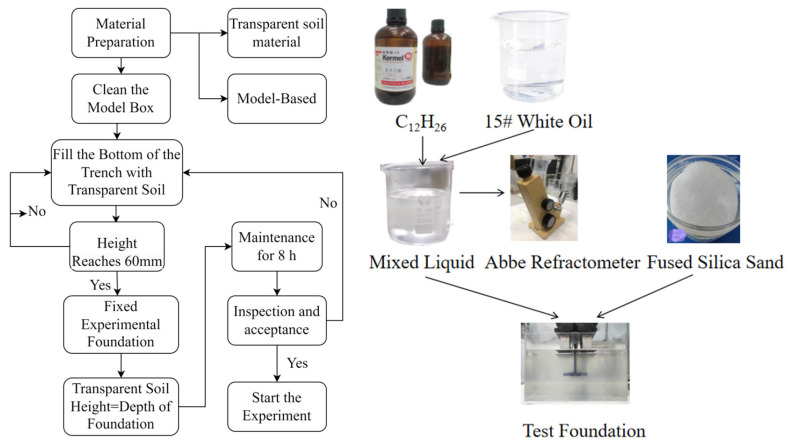
Flowchart and Model Foundation Pre-Embedding Process Diagram.

Based on transparent soil materials and particle image velocimetry (PIV) software, pull-out and horizontal tests were conducted on grillage root foundations of various sizes and with various root arrangements. The entire experimental process is divided into three key stages—model pile embedding, experimental execution, and image processing, as shown in Figure 3.

(1) Model Pile Embedding: Pour an appropriate amount of the pre-prepared mixture of C_12_H_26_ and No. 15 white oil into the model box. To ensure the overall transparency of the transparent soil, it is essential to observe the interaction between the foundation and the soil, confirming that the refractive indices of the mixed liquid and fused silica sand match. Next, add a small amount of quartz sand that does not exceed the liquid level of the transparent soil mixture. During the laying process, it is crucial to strictly control both the quantity of quartz sand added and the degree of leveling. Slowly stir the quartz sand and mixture with a glass rod to remove any air bubbles and achieve a nearly flat surface. Continue to add quartz sand and the mixed solution, stirring and leveling repeatedly until the quartz sand reaches the specified height.

(2) Experimental Development: Apply loads of varying directions and magnitudes to the test model foundation according to the predetermined loading plan to observe the deformation and failure processes of the test foundation. Simultaneously, high-frequency lasers and CCD high-speed industrial cameras are employed to record changes in the soil displacement field during the loading process.

(3) Image Processing Stage: In this stage, particle image velocimetry (PIV) software is used to track and process a series of laser speckle field images obtained during the experiment. Through software analysis and calculation, the complete displacement field of the soil surrounding the test foundation under load can be obtained, thereby further elucidating the bearing characteristics and failure mechanisms of the foundation.

## 3. Analysis of Experimental Results

In the model foundation setup, a pre-embedded installation approach was adopted to ensure a stable and reliable connection between the foundation and the model box. For the pull-out test, a stepwise loading method is implemented for control. The load increment for each level is set at 0.2 N, while the initial load is set at 0.4 N. During the loading process, the displacement of the foundation is continuously monitored until it is destroyed, at which point loading is stopped to determine the uplift bearing capacity of the foundation. In the horizontal loading test, different loading values are used for various types of foundations. For traditional foundations, the load increment for each level is set at 0.05 N, and the initial load is 0.1 N. For grillage root foundations, due to their structural characteristics, the load increment per level is increased to 0.1 N, while the initial load is set at 0.2 N. Throughout the loading process, the horizontal displacement of the foundation is continuously monitored until it is destroyed, at which point the failure criteria are established, and loading is terminated. The specific working conditions of the foundation are presented in Table 1.

### 3.1. Pull-Up Test Results

**Figure 4:**
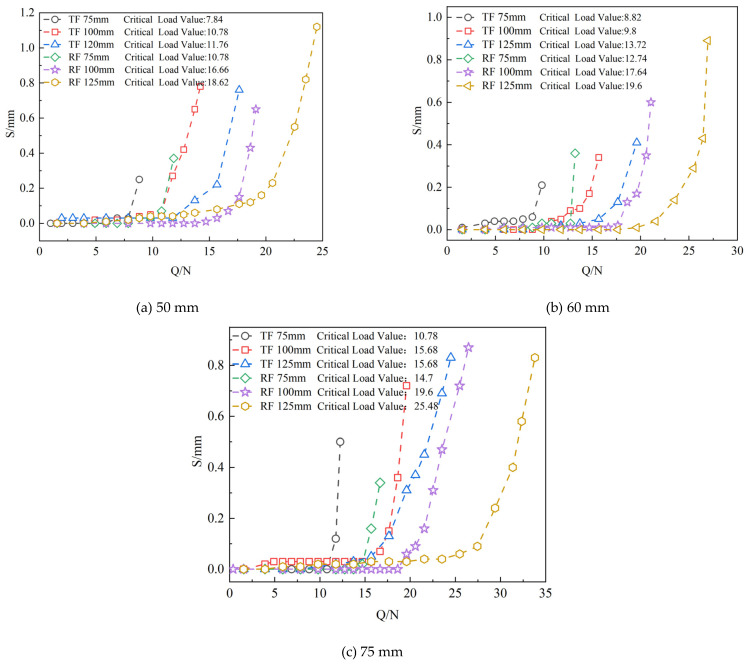
*Q-S* curves of foundations of different sizes and at different burial depths. Plate size: (**a**) 50 mm; (**b**) 60 mm; (**c**) 75 mm.

Conduct transparent soil model tests under vertical loading conditions on six different burial depths and sizes of grillage foundations and grillage root foundations using transparent soil materials. During the experiment, the upper-level load, prior to the foundation being pulled out, is defined as the ultimate load, which facilitates the accurate determination of the foundation’s pull-out bearing capacity. These experiments yield the load–displacement (Q-S) relationship curve, as shown in Figure 4. The comparative data of drawing and uplift bearing capacity on various foundations are shown in Table 2.

In the Figure 4, TF represents the grillage foundation, RF denotes the grillage root foundation, and the numbers indicate the burial depth of the test foundation. In the initial stage, when the load is relatively low, the change in displacement remains stable as the load increases. During this phase, the load supported by the top of the foundation is minimal. The uplift bearing capacity of the experimental foundation primarily relies on the self-weight of the upper soil for support, ensuring that the foundation structure remains stable under external loads. As the load increases further, the curve’s development pattern begins to change significantly, with displacement exhibiting varying degrees of nonlinear trends. Displacement shows a marked increase, continuing until the stage of structural failure. However, for grillage root foundations, the presence of roots effectively increases the foundation’s surface area and enhances the variable cross-sectional resistance. The grillage foundation initially displays a nonlinear growth trend, while the roots implanted around the foundation base plate significantly inhibit displacement development at the top of the foundation, thereby improving its bearing capacity.

**Figure 5:**
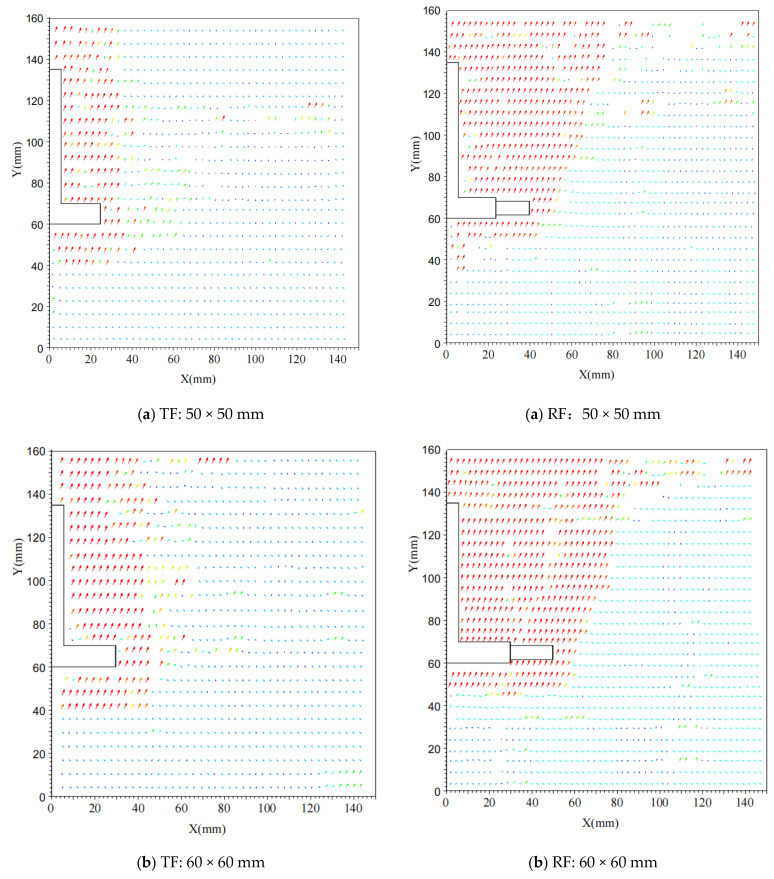

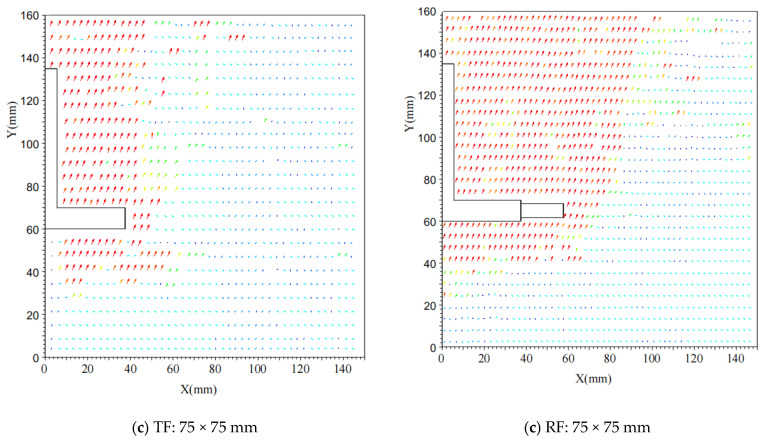
Distribution of Upward Displacement Field at 75 mm Burial Depth for Foundations of Different Sizes. Plate size: (**a**) 50 mm; (**b**) 60 mm; (**c**) 75 mm.

During the process of pulling up the grillage root foundation, as the vertical load is gradually applied, the foundation begins to experience upward displacement, causing the surrounding soil to shift accordingly. Due to the presence of the roots, the displacement of the soil around the roots becomes particularly pronounced, creating a distinct displacement movement area and forming a complex displacement field pattern. For analysis, consider the displacement field distribution diagram of different-sized foundations at a burial depth of 75 mm, as shown in Figure 5.

Figure 5 shows the distribution of the upward displacement field at a burial depth of 75 mm for foundations of different sizes. The vector arrows of the displacement field in the figure represent the magnitude and direction of the displacement at each point in the soil. The longer the arrow, the greater the displacement at that point; the direction of the arrow indicates the direction of displacement. The background color gradually changes from blue to red, indicating that the displacement amplitude increases from small to large. The blue area represents the area with a smaller displacement, while the red area represents the area with a larger displacement. This color gradient helps to visually observe the distribution and changes in displacement fields. In further analysis of Figure 5, firstly, from an overall perspective, as the foundation size increases, the range and intensity of the displacement field in the soil surrounding the test foundation show a significant upward trend. For a 50 mm base plate foundation, the displacement field of the surrounding soil is primarily concentrated in the area close to the foundation, exhibiting relatively small displacement vectors and a uniform distribution. This indicates that the disturbance and displacement of the soil are minimal during the uplift process of a smaller foundation. As the base size increases to 60 mm and 75 mm, the range of the displacement field in the surrounding soil expands significantly, with larger foundations causing greater disturbance and displacement effects on the surrounding soil when pulled up. Further observation reveals notable differences in the distribution patterns of displacement fields around different types of foundations. For grillage foundations, the displacement field primarily exhibits a rectangular distribution centered on the foundation, with the displacement vector direction being relatively consistent and mainly pointing away from the foundation. This indicates that the soil generates upward displacement around the foundation to resist the uplift force. In contrast, for grillage root foundations, the distribution pattern of the soil displacement field around the foundation primarily follows a parabolic shape. The roots act as a bridge connecting the foundation and the soil, effectively transferring the uplift load to the surrounding undisturbed soil, thereby enhancing the foundation’s pull-out bearing capacity. This also significantly alters the distribution and evolution of the displacement field of the soil around the foundation, resulting in more pronounced compression and tension effects in the displacement field around the roots. As the load gradually increases, the role of the root bond in the structure becomes increasingly prominent. Through its frictional interaction with the surrounding undisturbed soil, the load is effectively transmitted and dispersed over a wider area of undisturbed soil, further expanding the range and intensity of the displacement field.

### 3.2. Horizontal Test Results

**Figure 6:**
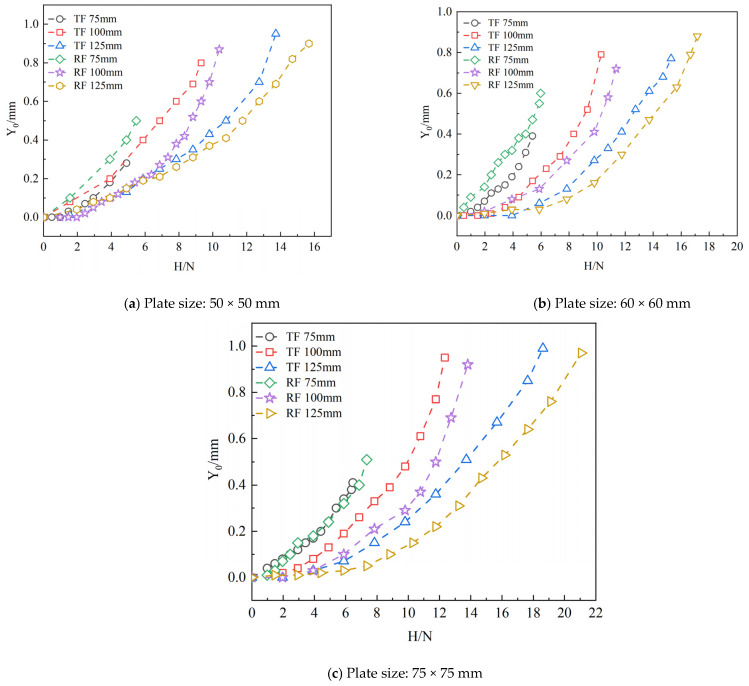
*H-Y*_0_ curves of foundations of different sizes and at different burial depths. Plate size: (**a**) 50 mm; (**b**) 60 mm; (**c**) 75 mm.

The comparative data of horizontal bearing capacity on various foundations are shown in Table 3. As shown in Figure 6, the horizontal load displacement (*H-Y*_0_) curves for six different sizes of grillage foundations and grillage root foundations (base plate sizes of 50 mm, 60 mm, and 75 mm) at various burial depths (75 mm, 100 mm, and 125 mm) are presented. Observing Figure 6, it is evident that when the load is small, the change in displacement is relatively minor, and the stiffness of the foundation is high, allowing it to resist a certain degree of horizontal force. As the load gradually increases, each additional horizontal load results in an increase in foundation displacement, with the increasing trend becoming more pronounced. The results indicate that when the load is small, the structure maintains a degree of stiffness, enabling it to withstand horizontal forces without significant displacement. However, once the load exceeds a certain critical value, the stiffness of the structure begins to diminish, leading to significant horizontal displacement. At this point, the foundation enters a nonlinear working state and may approach its ultimate horizontal bearing capacity. Further analysis of the horizontal load displacement curve in Figure 6 indicates that the size and burial depth of the base plate significantly impact the stiffness and ultimate horizontal bearing capacity of the grillage foundation. From a stiffness perspective, for grillage foundations of the same size, the initial stiffness of the structure increases with greater burial depth. Regarding ultimate horizontal bearing capacity, as the size of the base plate and the depth of the foundation increase, the ultimate horizontal bearing capacity improves significantly. For the grillage root foundation, the presence of the root enhances its stiffness and ultimate horizontal bearing capacity compared to the standard grillage foundation. The root effectively improves pile-soil interaction by increasing the contact area and friction between the foundation and soil, thereby enhancing the foundation’s bearing capacity. Under the same load conditions, the displacement of the grillage root foundation is significantly smaller than that of the grillage foundation, indicating that the root system effectively enhances the foundation’s ability to resist lateral displacement.

The failure of grillage foundations often occurs at the contact interface between the foundation base plate and the soil. Due to the low shear strength of the soil at this interface, when the horizontal load exceeds the shear strength, the soil undergoes shear failure, causing the foundation slab to lose support and ultimately fail. In contrast, under the same load conditions, the displacement of the grillage root foundation is significantly smaller than that of the grillage foundation. This is because the presence of roots greatly enhances the interaction between the foundation and the surrounding soil, demonstrating effective lateral displacement resistance. At the initial stage of loading, the roots effectively disperse and transmit horizontal loads by forming a tight embedding effect with the surrounding soil, thereby improving the safety of the overall structure. As the load gradually increases, the interaction between the foundation and the soil approaches its ultimate state. The root bond may sustain damage due to bending or shear action, resulting in a weakened or lost anchoring effect between the roots and the soil. Additionally, the presence of root bonds complicates the contact interface between the foundation slab and the soil, potentially leading to local shear failure or plastic deformation of the soil at this interface. The failure of grillage root foundations often occurs at the interface between the roots and the soil or within the roots themselves. When the horizontal load exceeds the shear bearing capacity of the roots, they may experience bending or shear failure, leading to a weakened or lost anchoring effect with the soil.

**Figure 7:**
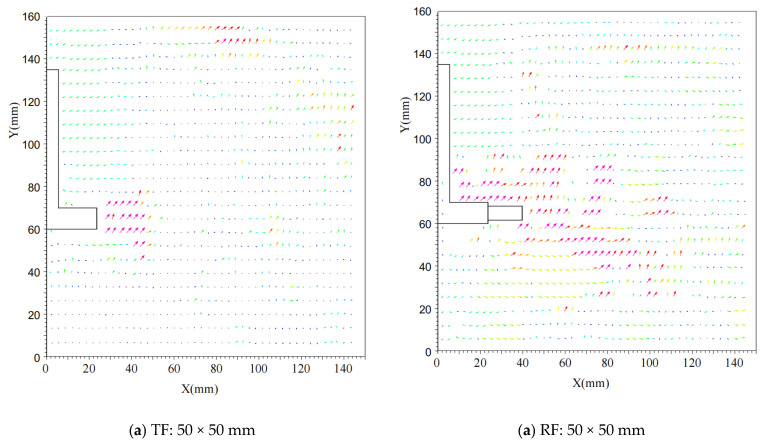

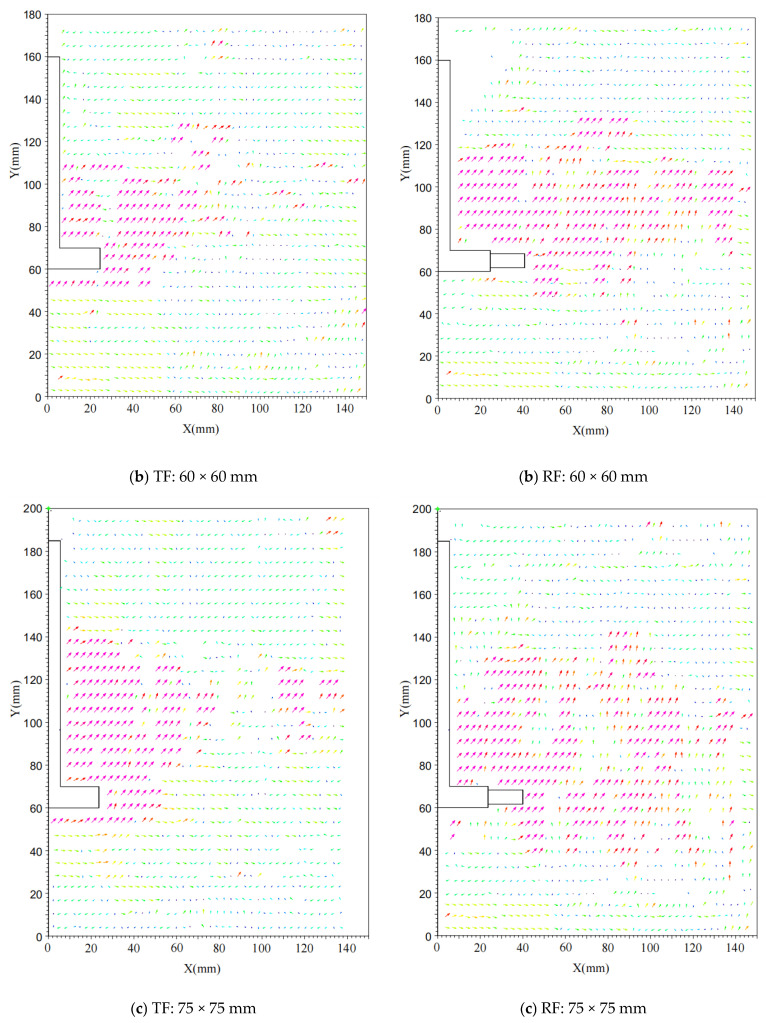
Distribution of Horizontal Displacement Field at 75 mm Burial Depth for Foundations of Different Sizes. Plate size: (**a**) 50 mm; (**b**) 60 mm; (**c**) 75 mm.

By processing the images captured by the camera, displacement fields for six different foundations under horizontal loads were obtained. As shown in Figure 7, the displacement field distribution of the test foundation under ultimate horizontal load is presented. The displacement field distribution diagram allows for a visual observation of the motion trajectory of the foundation under extreme conditions.

By comparing and analyzing Figure 7, it can be observed that, at different burial depths, the displacement field exhibits rigid rotational behavior around a specific point of the foundation support. For grillage foundations, the displacement vector direction generally points away from the foundation. Damage often occurs at the contact interface between the foundation slab and the soil, leading to shear failure of the soil and potential tilting or collapse of the foundation. In contrast, for grillage root foundations, the presence of roots induces compression and tension effects in the surrounding soil, resulting in a significant displacement gradient. The roots act as a bridge connecting the foundation and the soil, effectively transferring horizontal loads to the surrounding undisturbed soil. The range of the soil displacement field experiences notable changes due to the presence of root bonds. When subjected to the ultimate load, the displacement field of the soil around the grillage root foundation has a broader range of influence compared to the grillage foundation, and the variation in the displacement field around the foundation is more complex, especially in the vicinity of the roots. The implantation of roots not only alters the distribution of the soil displacement field around the foundation but also allows the soil in front of the foundation to mobilize a wider area to resist horizontal loads. Additionally, the soil behind the foundation can be effectively mobilized to resist horizontal loads due to the reverse rotation of the roots. This illustrates the significant advantage of roots in enhancing the overall load-bearing capacity of the foundation.

## 4. Machine Learning

The neural network method is a computational model that simulates the human brain’s nervous system, leveraging advancements in computer technology. It consists of a large number of artificial neurons interconnected to process complex information and make decisions, and it is employed across various fields. This article introduces the Elman model for predicting the bearing capacity of grillage root foundations and proposes the use of genetic algorithm optimization and swarm intelligence algorithms to find the optimal solution. This approach avoids the influence of random assignments on the prediction results, thereby improving the performance of the Elman neural networks.

### 4.1. Elman Model

The mathematical expression of the Elman neural network reflects its network structure and working principle. The basic mathematical model is presented in Equations (1) to (3):

(1) $$y(k)=g\left(W_{3}h(k)\right)$$

(2) $$h(k)=f\left(W_{1}c(k)+W_{2}u(k)\right)$$

(3) c(k)=αc(k−1)+(1−α)h(k−1)

In the formula, *y*(*k*) represents the output of the network at time *k*. g(⋅) is the transfer function representing the output layer neurons is usually a linear function; *W*_3_ represents the connection weight matrix from the hidden layer to the output layer; *h*(*k*) represents the output of the hidden layer of the network at time *k*; f(⋅) is the transfer function representing the hidden layer neurons and is usually a Sigmoid function or other nonlinear function; *W*_1_ represents the connection weight matrix from the context layer to the hidden layer; *c*(*k*) represents the output of the network’s receiving layer (context layer) at time *k*, used to store the output state of the hidden layer at the previous time; *W_2_* represents the connection weight matrix from the input layer to the hidden layer; *u*(*k*) represents the input of the network at time *k*; *α* is the self-connected feedback gain factor used to control the degree of memory of the output state of the hidden layer at the previous moment by the receiving layer, and its value range is usually between 0 and 1; *c*(*k* − 1) represents the output of the receiving layer in the network at time *k* − 1; *h*(*k* − 1) represents the output of the hidden layer of the network at time *k* − 1. From Equation (3), it can be seen that the feedback factor α is a parameter that needs to be selected in the Elman neural network, and the quality of its selection directly affects the output. In the following text, the genetic algorithm GA will be used to optimize α.

### 4.2. Elman Model Optimization Algorithm

The GA (genetic algorithm) is an optimization algorithm that simulates natural selection and genetic mechanisms. In the optimization process of the Elman neural network, the GA avoids local minima through selection operation, crossover operation, and mutation operation, thereby improving prediction ability. In 1975, Professor J. H. Holland from the University of Michigan clarified the theoretical model of genetic algorithms and explained their principles and characteristics. During the 1970s, K. A. De Jong conducted extensive numerical function optimization experiments on computers based on the idea of genetic algorithms. In the 1980s, D. J. Goldberg summarized and concluded findings from a series of research works. The running process of a genetic algorithm is iterative and generally includes the following five main steps: (1) randomly generating the initial population; (2) evaluating chromosomes using fitness functions; (3) selecting chromosomes with high fitness values to enter the next generation; (4) generating new chromosomes through genetic and mutation operations; and (5) repeating steps (2) to (4) continuously until the predetermined evolutionary generation is reached.

The WOA (whale optimization algorithm) is an optimization algorithm based on swarm intelligence. The WOA continuously adjusts network parameters and explores different regions in the parameter space to find the parameter combination that minimizes prediction error. This global search capability enables the WOA Elman model to more accurately capture patterns and patterns in complex datasets than standard Elman neural networks, thereby improving prediction accuracy. The WOA Elman neural network consists of three parts—the Elman neural network topology, the whale optimization algorithm, and the Elman neural network prediction. The whale optimization algorithm continuously optimizes the initial weights and thresholds of the network, enhancing the global optimization capability of the Elman neural network and preventing it from becoming stuck in local optima.

The Elman neural network, as a universal artificial neural network model, has a wide range of applicability. It can establish predictive models by learning the relationship between input data and output data, without relying on specific geological conditions or soil behavior. This article employs the genetic algorithm (GA) [30,31] and the whale optimization algorithm (WOA) [32] to optimize the Elman neural network, addressing the issue of neural network algorithms becoming trapped in local optima, with a focus on optimizing the initial weights and thresholds of the Elman network. To explore the response relationship between the load-bearing capacity of the grillage root foundation and the variation in influencing variables, these variables were used as research samples to construct training and testing sets. This approach aims to predict changes under conditions of foundation uplift and horizontal loading, respectively. Twelve sets of samples from different loading directions were selected for training, while six sets were reserved as the test set to construct the GA Elman and WOA Elman models, effectively predicting changes in basic bearing capacity.

The analysis was conducted using Python 3.9, an open-source software, through the input of relevant programs and data. The key advantages of Python include its concise and readable syntax, robust cross-platform compatibility, extensive third-party library ecosystem, and broad applicability across diverse domains. The computational results are presented in Section 4.3.

### 4.3. Result Analysis

**Figure 8:**
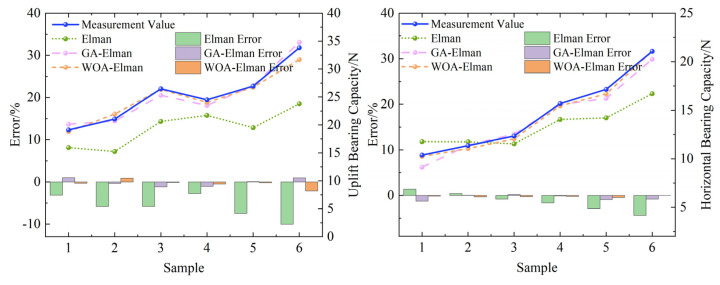
Comparison of Prediction Results.

When establishing a neural network prediction model, four physical indicators that affect foundation bearing capacity—namely, base plate size, burial depth, root length, and depth-to-diameter ratio—are used as input layer parameters, while the foundation bearing capacity serves as the output layer function. Based on the physical model experiment, the basic bearing capacity under different working conditions was obtained. Eighteen sets of samples were formed under uplift and horizontal loading conditions, consisting of 12 training samples and 6 testing samples. The number of input layer nodes was set to five, and the number of output layer nodes was set to one. The maximum training frequency was set to 1000, the learning rate to 0.01, and the minimum training target error to 1 × 10^−6^. The predicted results and errors are shown in Figure 8.

The trained prediction model also demonstrates the ability to predict basic bearing capacity, with the error requirement controlled within 10%. The results of the model prediction experiments meet these requirements, as shown in Figure 8. The solid line represents the actual value of the horizontal carrying capacity based on the foundation, while the dashed line represents the predicted value. The horizontal axis indicates the validation sample number. As illustrated in the figure, the distance between the optimized three lines is minimal, indicating that the difference between the predicted and actual values is small, which suggests that the optimized prediction model has excellent predictive ability. The comparison of evaluation indicators between the GA Elman model, WOA Elman model, and Elman model test set is shown in Table 4.

By analyzing the prediction results in Figure 8 and Table 4, we can conclude that: (1) all three models effectively demonstrate the trend of changes in basic bearing capacity, with relatively small errors compared to the monitoring values; (2) the WOA Elman model exhibits the smallest relative error percentage, as the Elman neural network has been optimized using the GA, effectively avoiding the disadvantage of becoming stuck in local minima and improving prediction accuracy. The relative average error of the GA Elman neural network in different loading directions is controlled within 5%, indicating that both models exhibit good prediction accuracy. The GA Elman model has high prediction accuracy and can better predict basic bearing capacity. The prediction curve of the test set shows that the predicted values of the WOA Elman neural network are significantly closer to the measured values. In terms of prediction error, the Elman neural network shows a relatively large prediction error, while the absolute prediction error of the WOA Elman neural network is almost always less than 1. Over 90% of the predicted values have an error of less than 0.5, indicating low prediction error and high prediction accuracy.

## 5. Conclusions

Based on transparent soil model experiments and machine learning methods, this study on the bearing performance of grillage root foundations has reached the following conclusions:

(1) The root structure significantly enhances the bearing capacity of the foundation, and the grillage root foundation effectively transfers and disperses loads through the interaction between the root and the soil. Experiments have shown that, compared to traditional foundations, the pull-out bearing capacity of the root foundation increases by 34.35% to 38.89%, while the horizontal bearing capacity improves by 10.76% to 14.29%.
(2) The basic size and burial depth significantly impact the mechanical properties, and increasing either the size or burial depth of the bottom plate greatly enhances the foundation’s resistance to uplift and horizontal bearing capacity. For example, the ultimate uplift bearing capacity of a 75 mm base-plate foundation (33.81 N) is 38% higher than that of a 50 mm foundation (24.5 N). When the burial depth increases from 75 mm to 125 mm, the uplift bearing capacity rises by as much as 41.5%.
(3) Under external loads, the root increases the contact area and friction between the foundation and the soil, utilizing the surrounding undisturbed soil to effectively transmit and disperse external loads, thereby enhancing the foundation’s bearing capacity. Through transparent soil material model experiments and particle image velocimetry (PIV) technology, visualization of the internal displacement field of the soil was achieved, verifying the optimization effect of the root structure on the soil displacement field.
(4) The Elman neural network was optimized using the genetic algorithm (GA) and the whale optimization algorithm (WOA), effectively addressing the issue of the model becoming stuck in local optima. The WOA Elman model has a prediction error controlled within 3%, which is superior to the traditional Elman model (with an error of up to 10%), providing a new method for high-precision prediction of complex soil-structure interactions.

For industrial practice, this discovery means that under the same geological conditions or stress conditions, using grillage root foundations can reduce foundation size or burial depth, thereby reducing construction costs. For academic research, this study demonstrates the enormous potential of transparent soil materials in simulating and analyzing the bearing characteristics of complex foundations, providing a new perspective for a deeper understanding of foundation bearing mechanisms and failure modes. Finally, by optimizing the Elman neural network model, high-precision prediction of the bearing capacity of the grillage root foundation was achieved, providing strong technical support for the design and construction of foundation engineering, and laying the foundation for the development of intelligent foundation engineering in the future.

## Figures and Tables

**Table 1:** Foundation Dimensions.

Base Plate Size/mm	Bracket/mm	Depth/mm	Root Length/mm	Total Number of Roots
50 × 50	130	75 100 125	—	—
50 × 50	130		15	8
60 × 60	130		—	—
60 × 60	130		20	12
75 × 75	130		—	—
75 × 75	130		20	12

**Table 2:** Comparison of Pulling Capacity on Various Foundations.

Base Plate Size/mm	Foundation Type	Depth/mm	Uplift Bearing Capacity/N	Increase Compared to TF/%
50 × 50	TF	75	8.82	—
		100	14.21	
		125	17.64	
	RF	75	11.85	34.35
		100	19.11	34.48
		125	24.5	38.89
60 × 60	TF	75	9.8	—
		100	15.68	
		125	19.6	
	RF	75	13.23	35.00
		100	21.07	34.38
		125	26.95	37.50
75 × 75	TF	75	12.25	—
		100	19.6	
		125	24.5	
	RF	75	16.66	36.00
		100	26.46	35.00
		125	33.81	38.00

**Table 3:** Comparison of horizontal bearing capacity of various foundations.

Base Plate Size/mm	Foundation Type	Depth/mm	Horizontal Bearing Capacity/N	Increase Compared to TF/%
50 × 50	TF	75	4.9	—
		100	9.31	
		125	13.72	
	RF	75	5.48	11.84
		100	10.38	11.49
		125	15.68	14.29
60 × 60	TF	75	5.39	—
		100	10.29	
		125	15.28	
	RF	75	5.97	10.76
		100	11.36	10.40
		125	17.15	12.24
75 × 75	TF	75	6.46	—
		100	12.34	
		125	18.62	
	RF	75	7.35	13.78
		100	13.81	11.91
		125	21.07	13.16

**Table 4:** Comparison of Predicted Data.

		Measurement Value/N	Elman Predicted Value	Elman Error	GA–Elman Predicted Value	GA– Elman Error	WOA–Elman Predicted Value	WOA– Elman Error
1	Uplift	19.11	15.9561	−3.1539	20.1364	1.0264	18.8093	−0.3007
2		21.07	15.2503	−5.8197	20.7206	−0.3494	21.9598	0.8898
3		26.46	20.6359	−5.8241	25.3014	−1.1586	26.3609	−0.0991
4		24.5	21.7289	−2.7711	23.4788	−1.0212	24.0187	−0.4813
5		26.95	19.5145	−7.4355	27.0102	0.0602	26.7418	−0.2082
6		33.81	23.7747	−10.0353	34.7861	0.9761	31.6929	−2.1171
1	Horizontal	10.38	11.7576	1.3776	9.124	−1.256	10.2445	−0.1355
2		11.36	11.7474	0.3874	11.3977	0.0377	11.0531	−0.3069
3		12.34	11.5387	−0.8013	12.5326	0.1926	12.0495	−0.2905
4		15.68	14.0527	−1.6273	15.5703	−0.1097	15.4478	−0.2322
5		17.15	14.2167	−2.9333	16.2236	−0.9264	16.6813	−0.4687
6		21.07	16.7017	−4.3683	20.2525	−0.8175	21.0691	−0.0009

## Data Availability

The data used to support the findings of this study are available from the corresponding author upon request.
